# Substance Use Attenuates Physiological Responses Associated With PTSD among Individuals with Co-Morbid PTSD and SUDs

**DOI:** 10.4172/2161-0487.S7-006

**Published:** 2013-08-30

**Authors:** Telsie A Davis, Tanja Jovanovic, Seth Davin Norrholm, Ebony M Glover, Mahogany Swanson, Sarah Spann, Bekh Bradley

**Affiliations:** 1 Department of Psychiatry and Behavioral Sciences, Emory University School of Medicine, USA; 2 Mental Health Service, Atlanta VA Medical Center, USA; 3 Department of Counseling and Psychological Services, Georgia State University, USA

**Keywords:** PTSD, Substance abuse, Hyperarousal, Fear responding, Acoustic startle

## Abstract

Posttraumatic stress disorder (PTSD) is often conceptualized from a fear conditioning perspective given individuals with PTSD demonstrate a reduced ability to inhibit fear even under safe conditions as compared to those without PTSD. The self-medication hypothesis suggests that individuals with PTSD often develop substance use disorders (SUDs) as an attempt to mitigate trauma-related distressing emotions. This investigation examined this hypothesis in a sample 214 participants, of which 81 did not meet criteria for either PTSD or SUDs (No diagnosis Control group); 33 met criteria for lifetime PTSD, but not SUDs (PTSD only group); 54 met criteria for lifetime SUDs, but not PTSD (SUDs only group); and 46 met lifetime criteria for both disorders (PTSD+SUDs group). PTSD was assessed using the modified PTSD Symptoms Scale (mPSS), SUDs were assessed using the Structured Clinical Interview for DSM-IV-TR (SCID). The startle magnitude was assessed using electromyography (EMG) of the eyeblink muscle in response to an acoustic startle probe. Fear-potentiated startle (FPS) was analyzed by comparing startle magnitude at baseline to startle during a fear conditioned stimulus. Results showed that PTSD significantly increased startle responses. However, there was a significant effect of SUDs on fear-potentiated startle to the danger signal, in that those who met criteria for SUDs had reduced fear compared to those who did not. The individuals who had co-morbid PTSD and SUDs did not differ from the Control group. Findings indicate that SUDs may attenuate exaggerated fear responses associated with PTSD. Consistent with the self-medication hypothesis, results suggest that substance use may co-occur with PTSD because it reduces heightened fear load and may allow normalized function in traumatized individuals.

## Introduction

There is a high degree of co-morbidity with substance use disorders (SUDs) among individuals with posttraumatic stress disorder (PTSD) [1–3]. Among individuals with a lifetime PTSD diagnosis, 51.9% of men and 27.9% of women also met lifetime criteria for alcohol abuse or dependence, and 34.5% of men and 26.9% of women also met lifetime criteria for drug abuse or dependence [1]. Conversely, among individuals with a lifetime diagnosis of alcohol abuse, 2.5% of men and 10.5% of women also had a lifetime diagnosis of PTSD [2]. For those with a lifetime diagnosis of alcohol dependence, the prevalence rate of a lifetime diagnosis of PTSD increased to 10.3% of men and 26.2% of women [3].

Individuals with co-morbid PTSD and SUDs are significantly more likely to have poorer treatment compliance, retention, and outcome in comparison to individuals with a single diagnosis of either disorder [4,5]. To address these problems, research studies and randomized controlled trials are being conducted and the numbers of investigations are increasing. However, evidence-based interventions to treat this co-morbidity remain few in number and questions of efficacy remain [4]. We suggest that a better understanding of the functional relationship between PTSD and SUDs could yield more nuanced treatment considerations that may ultimately improve treatment outcomes for individuals with this co-morbidity.

Much of the published data concerning the association between PTSD and SUDs is consistent with the widely-held “self-medication hypothesis,” which posits that individuals with PTSD intentionally use substances of abuse to alleviate or manage their trauma-related symptoms [6–8]. Substantial findings support a pathway whereby PTSD develops first [9,10]. The National Comorbidity Survey replication documented that the median age of onset for anxiety disorders is nine years earlier than for SUDs [11]. Additionally, and particularly for women, robust retrospective data show that problematic substance use develops in response to using substances to cope with the negative effects of trauma exposure [12–17]. Support for the self-medication hypothesis is strong, but not universal. As such, other hypotheses remain tenable.

Three other distinct hypotheses on the PTSD-SUDs link are well-documented in the literature. The “high-risk hypothesis” proposes that individuals with SUDs are at a greater risk of developing PTSD due to increased vulnerability to interpersonal violence (e.g., sexual assault) that ensues from their at-risk, substance-abusing lifestyle [16,18]. The “susceptibility hypothesis” suggests that individuals with SUDs have an increased vulnerability to developing PTSD due to genetic or psychological impairments that result from substance use [18,19]. The “cross-sensitization hypothesis” is derived from animal models of addictive behaviors (for a review see earlier work [20]) that have shown that exposure to stressors enhances psychostimulant-induced dopamine transmission along the mesocorticolimbic pathway and increases the locomotor [21,22] and rewarding [23,24] effects of stimulants such as cocaine. From a clinical perspective, this suggests that stress (i.e., trauma) primes the reward system such that when an individual uses substances of abuse, he or she becomes more susceptible to the rewarding effects of the drug(s), increasing the likelihood of the development of SUDs.

Clarity about the functional association between PTSD and SUDs is necessary to identify treatment targets specific to this co-morbidity. The competing hypotheses to date are likely due, at least in part, to the inability of psychological research strategies to capture direct empirical support [25]. Especially given that the majority of the psychological research is based on self-report data and cross-sectional designs that do not allow for the testing of causation [25]. We suggest that objective, psychophysiological measurement can advance our understanding about the functional association between PTSD and SUDs beyond that previously offered by psychological assessment solely reliant on self-report and cross-sectional data.

The fear-related symptoms of PTSD (e.g., re-experiencing/intrusion) are often conceptualized from a fear conditioning perspective given individuals with PTSD have been reported to exhibit impaired fear conditioning in comparison to those without PTSD [26]. This makes it possible to investigate PTSD using physiological measurements such as the acoustic startle response (ASR). Among individuals with PTSD, the degree of fear acquisition and fear expression is so robust that the prefrontal cortex is unable to inhibit exaggerated amygdala signaling [27]. This difficulty inhibiting fear responses is reflected in research on psychophysiological responses of individuals with PTSD, including increased levels of ASR [28].

A growing body of research supports the idea that substance use serves as an anxiety-regulation strategy employed by traumatized individuals to attenuate or control affective distress (e.g., fear and hyperarousal) associated with PTSD [8,25]. This idea is supported by psychophysiological studies in humans and other animal models showing an association of substance use with diminished ASR [29]. For example, chronic cocaine dependence in early remission [30] and opioid maintenance therapy [31] have been associated with significant reductions in ASR. Thus, we propose using psychophysiological measurement to investigate whether co-morbid substance use diminishes the increased ASR associated with PTSD.

The aim of this investigation was to use the acoustic startle paradigm to determine whether substance use functions as an anxiety-regulation strategy on a physiological level among individuals with co-morbid PTSD and SUDs. Based on: (1) past studies demonstrating greater fear-potentiated startle in PTSD subjects during fear acquisition and fear extinction (i.e., fear loading) in comparison to control subjects [32] and (2) robust data in support of the self-medication hypothesis identifying substance use as an emotion-regulation strategy, we hypothesized that individuals with co-morbid PTSD and SUDs will demonstrate a diminished fear response at baseline and conditioning in comparison to individuals with PTSD only, but a greater fear response in comparison to individuals with SUDs only or those without PTSD or SUDs. We also hypothesized that PTSD would be associated with higher fear responses to safety signals, based on our previous work with this disorder [28].

## Method

### Participants

The study included 214 participants, of which 81 did not meet criteria for either PTSD or SUDs (Control group), 33 met criteria for lifetime PTSD, but not SUDs (PTSD Only group), 54 met criteria for lifetime SUDs, but not PTSD (SUDs Only group), and 46 met criteria for lifetime diagnoses for both disorders (PTSD+SUDs group). Participants were recruited as part of a larger study examining the contribution of genetic and environmental factors to the development of PTSD among a largely African-American, low socioeconomic status, and inner-city population seeking non-psychiatric services at a large, urban Southeastern U.S. hospital. Exclusion criteria included active psychosis, hearing impairment, positive toxicology on a urine drug screen for cocaine and opiates, along with major medical illnesses as assessed through health and physical examinations conducted by licensed medical professionals. All participants were screened for auditory impairment using an audiometer (Grason-Stadler, Model GS1710) and were required to detect tones at 30 dB (A) SPL at frequencies between 250 to 4000 Hz. Before participation in the study, participants provided written informed consents approved by the Emory University Institutional Review Board and the Grady Health System Research Oversight Committee.

### Measures

#### Psychological measures

In addition to the diagnostic interview, all participants were administered six measures to assess specific psychological symptom presentations. The Trauma Experiences Inventory (TEI) is a 14-item self-report instrument used to assess the occurrence, intensity, and frequency of 13 different types of traumatic events across the life span [33]. It is a measure of both child and adult trauma exposure. The total frequency score was used in this investigation to control for level of lifetime trauma exposure.

The Childhood Trauma Questionnaire–Short Form (CTQ-SF) is a 28-item self-report inventory administered to measure childhood physical, sexual, and emotional abuse [34]. Empirical findings have established that the CTQ-SF has demonstrated good internal reliability and criterion validity and is psychometrically comparable to the original 70-item measure [34]. The CTQ-SF yields a total score, as well as a subscale score for each type of child maltreatment assessed. The total score was used as a covariate in this analysis.

The Beck Depression Inventory-II (BDI-II) is a 21-item questionnaire used to detect depressive symptoms and severity of those symptoms over the two-week period prior to assessment [35]. Items are measured on a scale of 0 to 3, and the total score was used as an indicator of depressive symptomatology in this study.

The Modified PTSD Symptoms Scale (mPSS) is a 17-item self-report scale used to assess PTSD symptom severity over the two-week period prior to rating [36]. The items on the mPSS mirror the DSM-IV-TR criteria for PTSD. The scale renders both a total score and a total score for each PTSD symptom cluster (i.e., re-experiencing, avoidance/numbness, and hyperarousal). Research indicates the mPSS demonstrates adequate internal consistency and good concurrent validity [36]. The mPSS total score and the total score for each symptom cluster were examined in this study.

The Structured Clinical Interview for DSM-IV-TR Axis I Disorders (SCID) is a clinician-administered, validated assessment measure administered to evaluate the presence or absence of SUDs within our study population [37].

The Kreek–McHugh–Schluger–Kellogg scale (KMSK) is self-report scale that assesses the amount, frequency, and duration of self-exposure to alcohol, opiates, cocaine, marijuana, and tobacco [38]. Lifetime use of substances as well as current use within the 30 days prior to assessment is measured. Lifetime substance use was examined in this study sample.

#### Startle response measurements

The eye blink component of the ASR was measured by electromyogram (EMG) recordings of the right orbicularis oculi muscle with two 5 mm disposable Ag/AgCl electrodes filled with electrolyte gel. One electrode was placed 1cm below the pupil of the right eye and the second was positioned 1cm below the lateral canthus. We used a Check trode impedance meter (1089 MKIII, UFI, Morro Bay, CA) to ensure impedance levels were less than 6 kOhm for each participant. The startle probe was a 108 dB (A) SPL, 40msec burst of broadband noise with near instantaneous rise time, delivered binaurally through headphones (Maico, TDH-39-P). Startle response data were acquired using Biopac MP150 for Windows (Biopac Systems, Inc., Aero Camino, CA) and stored on the hard drive of a Windows XP laptop computer. All data were sampled at 1,000 Hz and amplified with an increase of 5,000 using the EMG module of the Biopac system. MindWare software (MindWare Technologies, Ltd., Gahanna, OH) was used to filter, rectify, and smooth the startle response data, as well as export it for statistical analyses. The EMG signal was filtered with low- (28 Hz) and high-frequency (500 Hz) cut-offs, respectively. The maximum amplitude of the eye blink muscle contraction occurred 20–200 msecs after presentation of the startle probe, and it was used as a measure of the ASR.

The fear-potentiated startle task included two phases: habituation and fear conditioning. The habituation phase consisted of six startle probes presented alone (noise-alone trials, NA). Immediately following habituation, participants underwent the fear conditioning phase, which consisted of three blocks, each of which included four trials of the reinforced conditioned stimulus (CS+, “danger cue”), four trials of the non-reinforced conditioned stimulus (CS−, “safety cue”), and four NA trials for a total of 12 trials per block. All CS+ trials were reinforced with the aversive unconditioned stimulus (US), while the CS− trials were not reinforced. Both CS’s were different colored shapes presented on a computer monitor and were six seconds in duration. The US was a 250 ms air blast with an intensity of 140 psi directed to the larynx. The air blast was emitted by a compressed air tank attached to the polyethylene tubing and controlled by a solenoid switch. This US has been used in our studies previously [39,40] and produces robust fear-potentiated startle. In all phases of the experiment, inter-trial intervals were of randomized duration ranging from 9 to 22 seconds.

#### Cognitive awareness measurement

A response keypad unit (SuperLab, Cedrus, Corp., San Pedro, CA) was incorporated into the startle session in order to assess trial-by-trial US-expectancy and contingency awareness [41]. Subjects were instructed to respond on each CS trial, within 3 seconds of CS onset, by pressing one of three buttons: a button marked ‘+’ when they expected the US, a second button marked “−” when they did not expect the US, and a third button marked ‘0’ when they were uncertain of the contingency. The exact instructions given to the subjects were: “During this experiment you will hear some sudden tones and noises in addition to seeing several colored shapes on the computer monitor. The noises are there to elicit startle and occur every time something happens. However, some of the shapes will be followed by a blast of air while other shapes will not. Throughout the experiment please press the button on the keypad to tell us whether you think a shape will be followed by air (the plus sign), or will not be followed by air (the minus sign). If you do not know, press the 0 sign. You should press a button for each shape.”

### Data analysis

The group variables in the analyses were the diagnostic categories for PTSD and SUDs, resulting in two between-groups factors: SUD (2 levels) and PTSD (2 levels). This allowed for the analyses of main effects of each disorder as well as any interaction effects. We also followed up significant effects with Post-hoc comparison between four groups: No diagnosis Control, PTSD only, SUDs only, and co-morbid PTSD+SUDs.

The magnitude of the startle response across the three conditioning blocks and trial types (NA, CS+, CS−) was compared between the two factors using a mixed model 3 (Block)×3 (Trial Type)×2 (SUD)×2 (PTSD) analysis of variance (ANOVA). Contrasts were analyzed for Block (linear trend) and Trial Type (NA vs CS+, CS+ vs. CS−). In addition, in order to compare the degree of fear-potentiated startle between groups while controlling for individual variability in baseline startle responses (i.e., to the NA trials), we calculated a Difference Score by subtracting startle magnitude to the NA trials from the startle magnitude on CS+ trials and CS− trials in each conditioning block. These Difference Scores were also used as dependent variables in a mixed model ANOVA as described above. As mentioned, we compared between-group differences using a four group categorization (Control, PTSD only, SUDs only, and PTSD+SUDs). This categorization was also used for descriptive statistics to better compare the individual groups. Post-hoc group comparisons were performed using Tukey’s HSD tests. All statistical analyses were performed using SPSS 20 for Windows. For repeated-measures ANOVA, we used the Huynh-Feldt correction for degrees of freedom. Alpha was set at 0.05.

## Results

Demographic data for the four groups are shown in Table 1. As a whole, the average age was 39.97 years old (SD=11.78), primarily African American (92.6%), 66.0% female, and 78.6% were unemployed. There were significant group differences in age, F(3,214)=4.68, p=.003, sex, *χ^2^*(3)=24.14, *p*<.001, race, *χ^2^*(12)=20.91, *p*=0.05, and unemployment, *χ^2^*(3)=9.35, *p*<0.05. As shown in Table 1, the co-morbid PTSD+SUDs group was older than the PTSD Only group, the proportion of females was higher in the Control and PTSD Only groups, and lower in the SUDs Only group. In addition, the proportion of African American was highest in the Control group. Finally, participants in the Control group were less likely to be unemployed than those in the three diagnostic groups. Table 2 shows the clinical data across the four groups. The co-morbid PTSD+SUDs group had greater total PTSD symptoms as compared to the SUDs only and PTSD only group, and higher lifetime cocaine use than the SUDs only and PTSD only groups. Looking at PTSD symptom clusters, the co-morbid group had significantly higher avoidance and hyper arousal symptoms than all other 3 groups, while the re-experiencing symptoms were comparable to the PTSD only group and higher than the Control and SUDs only groups.

**Figure 1:**
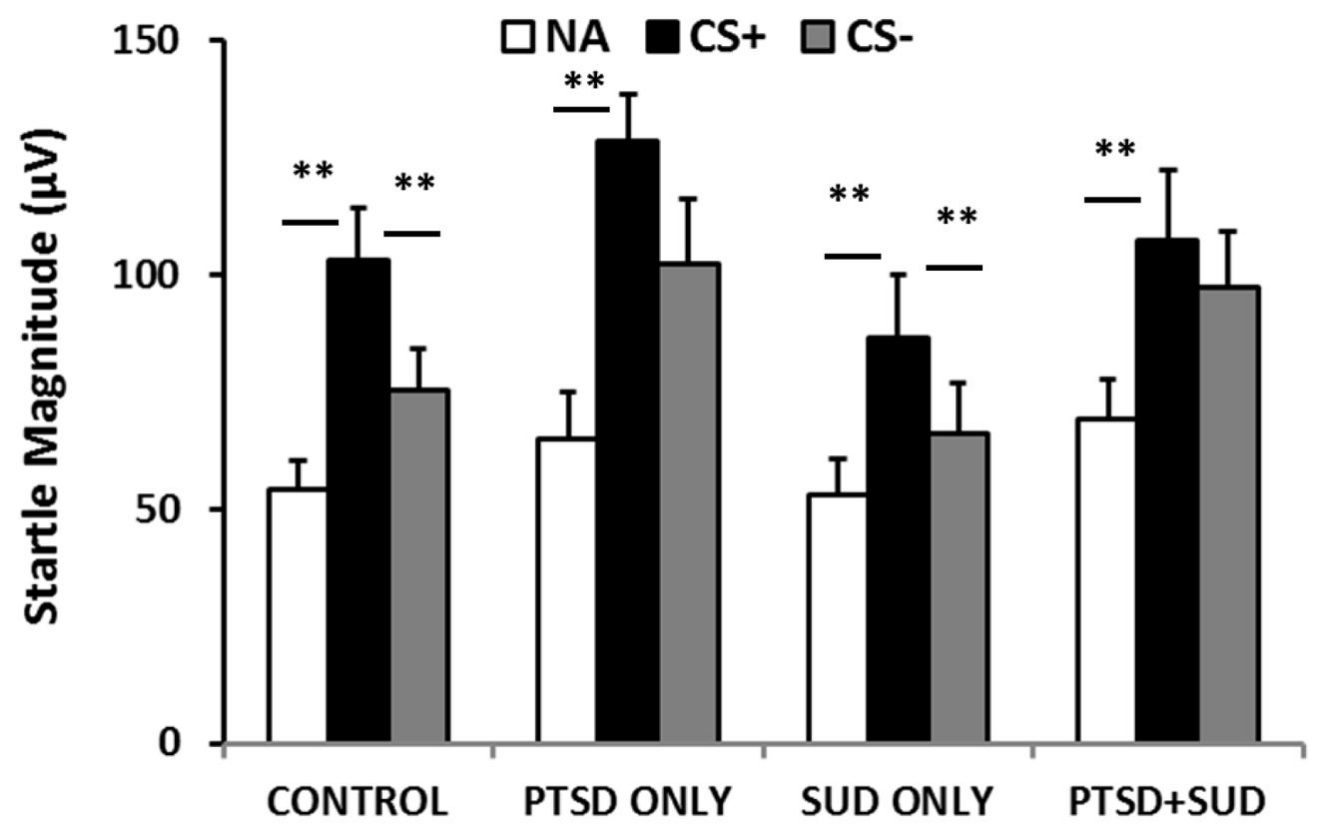
Mean ± SE startle magnitude during late fear conditioning across Trial Type and diagnostic groups. All groups show increased startle responses to the CS+ compared to the NA trials. Only the Control (neither diagnosis) and the SUD only groups show significantly higher startle to the CS+ than the CS−. **=p<.001. Abbreviations: CS+=reinforced conditioned stimulus; CS−=non-reinforced conditioned stimulus; NA=noise alone.

A mixed model 3×3×2×2 ANOVA of startle magnitude with Block (3 levels)×Trial Type (3 levels)×SUDs (2 levels)×PTSD (2 levels) as factors resulted in a significant interaction effect of Block×Trial Type×SUDs×PTSD, *F*(4, 836)=3.67, *p*=0.006. We followed up the interaction by examining the effect of Trial Type×SUDs×PTSD during the last two blocks of conditioning, when learning is maximal. Figure 1 shows the startle amplitude across Trial Types and diagnoses. We found a significant interaction of SUDs and Trial Type for the NA vs CS+ contrast, *F*(1, 210)=4.11, *p*<0.05, and a significant main effect of PTSD, *F*(1, 210)=4.29, *p*<0.05, with the PTSD subjects having higher startle responses compared to non-PTSD subjects. Although we did not find an interaction effect of SUDs with PTSD, we wanted to examine fear potentiation (NA vs. CS+), and discrimination (CS+ vs. CS−) in each diagnostic category separately given our hypotheses based on our previous findings in PTSD.

We compared the three trial types within each group and found that all four groups demonstrated successful fear conditioning with a significant increase in startle response to the CS+ compared to NA (Control, *F*(1,80)=32.51, *p*<0.001; PTSD only, *F*(1,32)=18.55, *p*<0.001; SUDs only, *F*(1,53)=22.60, *p*<0.001; PTSD+SUDs, *F*(1,45)=20.45, *p*<0.001). However, only the Control and SUDs only groups showed significantly higher startle response to the CS+ compared to CS− (Control, *F*(1,80)=20.11, *p*<0.001; SUDs only, *F*(1,53)=8.85, *p*=0.004), while neither of the PTSD groups did, (Figure 1). We repeated the above analysis while co-varying for age and found that the Control group still demonstrated significant fear conditioning to the CS+ and discrimination between the CS+ and CS−. The PTSD Only group showed significant fear conditioning to the CS+, but not discrimination. The last two groups no longer showed significant fear conditioning or discrimination.

**Figure 2:**
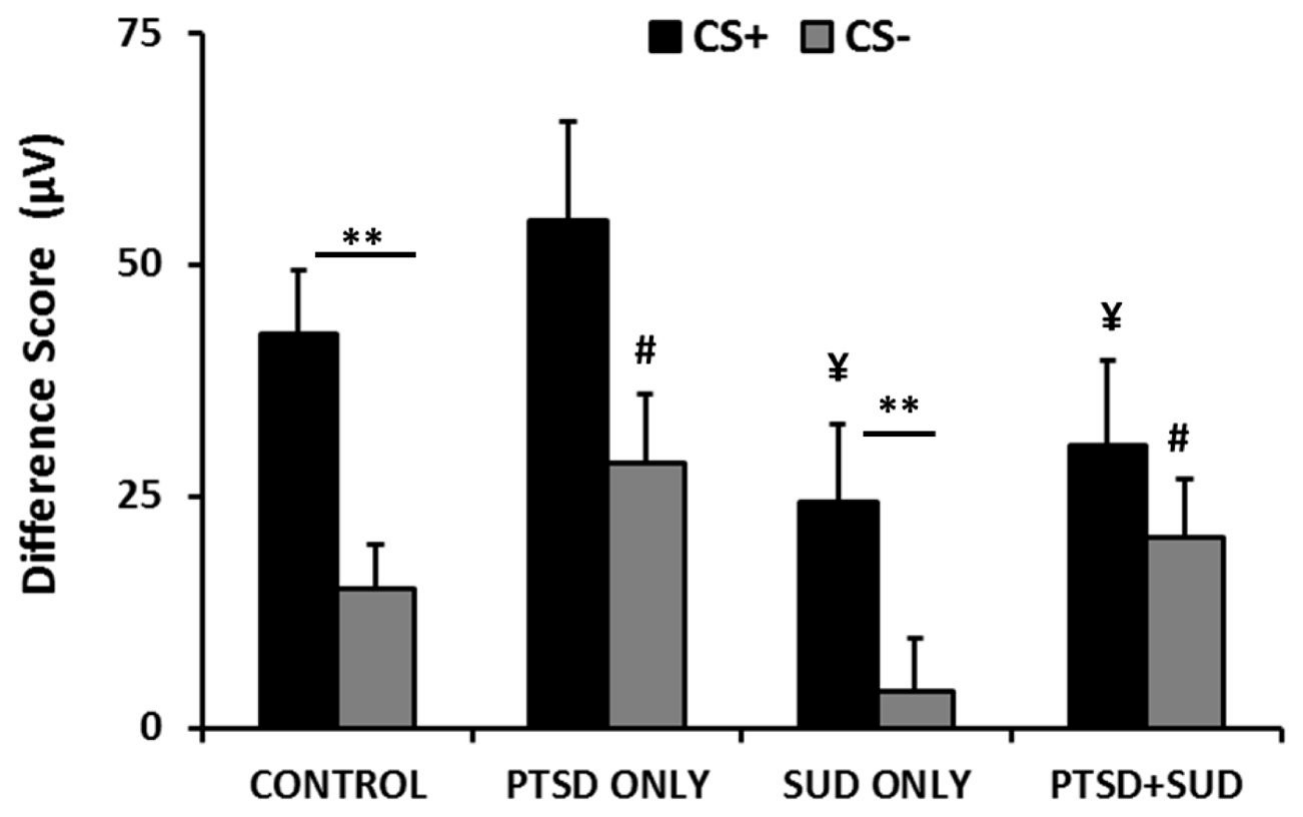
Mean ± SE difference score (calculated as the difference in startle magnitude between the CS and NA) during late fear conditioning across Trial Type and diagnostic groups. Only the Control (neither diagnosis) and the SUDs only groups show significantly higher startle to the CS+ than the CS−. The two SUDs groups have lower fear-potentiated startle to the CS+, and the two PTSD groups have higher startle to the CS−. **=p<.001; ¥=p<.05 main effect of SUD; #=p<.05 main effect of PTSD. Abbreviations: CS+=reinforced conditioned stimulus; CS−=non-reinforced conditioned stimulus.

In order to compare the degree of fear-potentiated startle between groups and control for individual differences in baseline startle, we calculated a Difference Score by subtracting startle responses to the NA from startle to each CS. We then again examined the Difference Score between CS+ and CS− during late conditioning within each diagnostic group. We again found that the Control and SUDs only group showed significant discrimination (*F*(1,80)=20.12, *p*<0.001, and *F*(1,53)=8.85, *p*<0.005, respectively), while the PTSD only and PTSD+ SUDs groups did not (Figure 2). However, after co-varying for age, the SUDs only group no longer showed significant discrimination. Given our hypotheses regarding safety signals, we examined the responses to danger cues and safety cues separately, and used each Difference Score as the dependent variable in a 2×2 ANOVA with SUDs (2 levels) and PTSD (2 levels) as between-groups variables. As shown in Figure 2, there was a main effect of SUDs on the fear-potentiated startle to the CS+, *F*(1,210)=5.74, *p*=0.02), in that those with substance disorders had reduced fear responses in both PTSD groups. With regard to the CS−, we found a main effect of PTSD, *F*(1,210)=6.13, *p*=0.01), in that those who met criteria for the diagnosis showed increased fear to the safety signal across both SUDs groups. We again repeated the analysis with age as the co-variate and the effects remained significant. Therefore, the comorbid SUDs+PTSD group had reduced levels of fear to the danger cue and increased fear to the safety cue, resulting in poor discrimination between the two stimuli.

**Figure 3:**
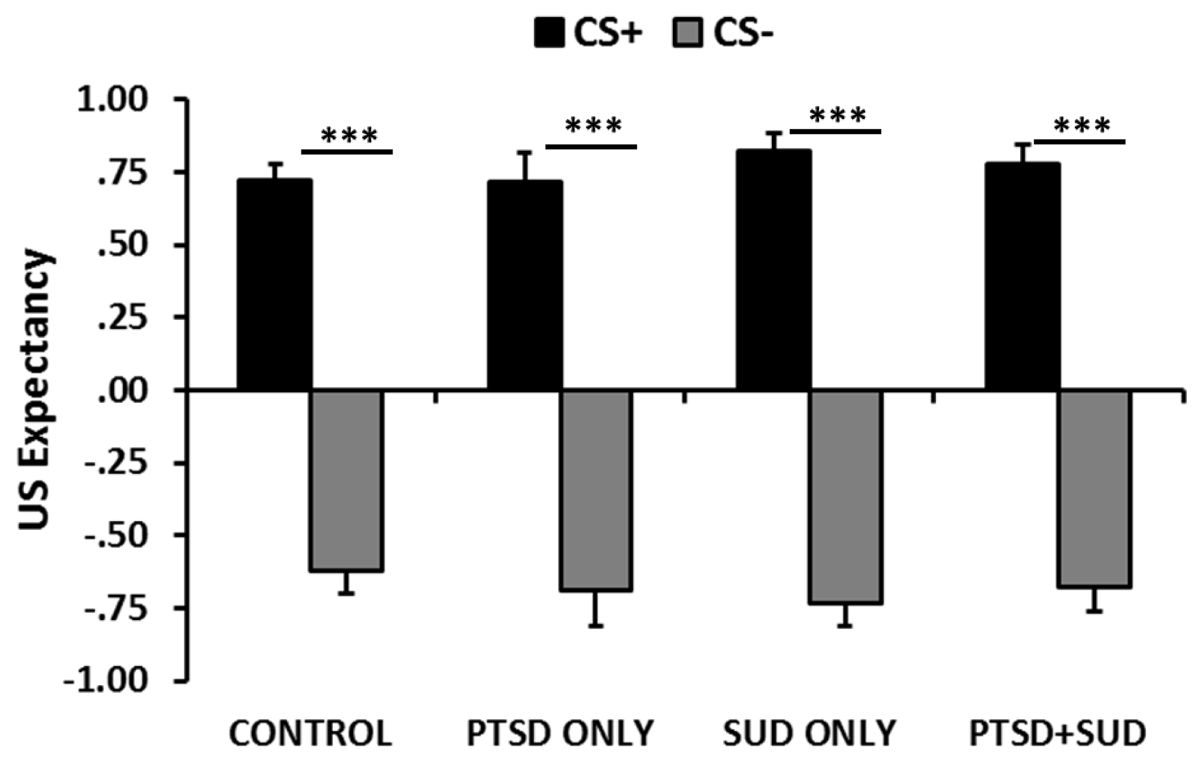
Mean ± SE US expectancy reported on the keypad during late fear conditioning across Trial Type and diagnostic groups. All groups showed significantly higher expectancy of the airblast on the CS+ the CS- trials. *** =p<.0001. Abbreviations: CS+=reinforced conditioned stimulus; CS−=non-reinforced conditioned stimulus.

In order to assess whether SUDs had an impact on cognitive awareness of the reinforcement contingencies in the fear conditioning task, we examined the data from the trial-by-trial US-expectancy responses for each CS. These data were available for a subset of participants, *n*=147 (Control, *n*=53; PTSD only, *n*=17; SUDs only, *n*=40; PTSD+SUDs, *n*=37) and were used to categorize participants as aware of the contingencies if they had an expectancy of the US on the CS+ trials, but no expectancy of the US on the CS- trials during the last conditioning block. This resulted in 108 aware participants (73%), equally distributed across the four groups, *χ^2^* = 4.21, p=0.24. We then used the US-expectancy in the aware participants as the dependent variable in a mixed model 3×2×2×2 ANOVA with Block×Trial Type× SUDs×PTSD as the factors, as described above. We found a significant two-way interaction of Block×Trial Type, *F*(2,204)=70.74, *p*<0.001, but no main or interaction effects of either SUDs or PTSD. Looking only at late blocks of conditioning, there was a significant effect of Trial Type, *F*(1,104)=355.77, *p*<0.0001, with higher US-expectancy on the CS+ than the CS−, (Figure 3). Again there was no significant main effect or interaction effect with SUDs or PTSD.

Finally, in order to see whether awareness of the experimental contingencies affected the startle results reported above, we compared the startle difference score for CS+ vs. CS− within each group, after removing unaware participants. The results remained the same. In other words, the Control and SUDs only groups showed significant discrimination between the CS+ and CS− on the fear-potentiated startle measures, while the two PTSD groups did not, even when they were cognitively aware of the contingencies. We also repeated the analyses of startle and response pad data after removing individuals that met current criteria (last 30 days) for SUDs on the SCID, *N*=15. The results did not change, indicating that chronic lifetime drug use was driving the observed SUDs results.

## Discussion

The purpose of the present study was to investigate, for the first time to our knowledge, whether an objective, psychophysiological measurement could provide information on the relationship between PTSD and SUDs that would shed light on the reason for their frequent co-occurrence. The findings resulted in two main results. First, we replicated our previous findings of heightened fear-potentiated startle in PTSD subjects as well as a lack of discrimination between danger and safety cues [28]. Second, we found that participants with SUDs had decreased levels of fear-potentiated startle to the danger cue (CS+) compared to the participants without SUDs, in the control and the PTSD only group. This result suggests that substance use attenuates exaggerated fear responding associated with PTSD.

Consistent with the self-medication hypothesis, our results suggest that substance use, in general, may co-occur with PTSD because it reduces heightened fear and allows for normalized function in traumatized individuals. Our previous studies with PTSD indicated that heightened fear responses, termed “fear load”, is associated with slower fear extinction [32] and increased attention bias to negative emotion [42]. High fear load may be one of the aspects of PTSD that results in significant dysfunction and maintenance of symptoms; and it is possible that substance use allows for a self-imposed reduction in fear load resulting in normalized function. It is important to note that the PTSD+SUDs group reported higher levels of clinical symptoms (PTSD total and avoidance and hyperarousal symptom clusters) than the PTSD only group; therefore, the attenuation appears to only alleviate physiological responses rather than self-report of symptoms.

As mentioned above, the ability to discriminate between danger (CS+) and safety (CS−) signals at the physiological level was found in both the Control and SUDs only groups, but not in either PTSD group. The PTSD only group showed high levels of fear on both cues, while the PTSD+SUDs co-morbid group showed less fear to both cues. It is possible that patients who suffer from co-morbid PTSD and SUDs are less fearful, but still unable to discriminate between danger and safety at the physiological level, increasing their likelihood of engaging in high-risk behaviors that predispose them to further trauma exposure. Although co-varying for age did not change the results in the PTSD groups, the SUDs only group no longer showed discrimination between danger and safety; therefore, it is possible that the impact of age on discrimination may explain the deficits in the comorbid group which was on average older than the other groups.

Importantly, results show that the lack of discrimination between cues is not due to cognitive impairments–even chronic substance use did not result in either higher rates of unaware participants or impaired cognitive learning. Furthermore, neither the PTSD only nor the PTSD+SUDs groups showed deficits in awareness or learning (Figure 3). Therefore, the discrimination deficits in these two groups were only associated with psychophysiological responses. These results indicate that there was a discrepancy in the cognitive and psychophysiological responses in the PTSD participants: even though they did not expect the US to be delivered on the CS-trials (Figure 3), they did not show lower levels of fear-potentiated startle compared to the CS+ (Figure 1 and 2). We have observed this dissociation between cognition and fear-potentiated startle responses in several previous studies with PTSD [26,28,32,43]. This dissociation may be due to deficient prefrontal cortex inhibition of amygdala activity in PTSD, which has been shown in neuroimaging studies [44].

Chronic cocaine dependence has been shown to have long-term effects on startle responses, in that cocaine users showed reduced startle even after a year of abstinence [30]. Acute cocaine use is associated with increased activity of the neurotransmitter dopamine (DA) in several brain areas associated with reward [45]. However, chronic cocaine use may result in adaptations in these brain areas, so that the long-term effects of cocaine are associated with lower levels of DA [46]. This neurotransmitter is also known to have effects on startle response by modulating the startle circuit via the amygdala and ventral striatum [47]. Our data support this hypothesis given that we found a main effect of SUDs on fear-potentiated startle to the CS+; it is possible that the hypodopaminergic state in the co-morbid group is the underlying neurobiological mechanism for the reduction in startle responses. On the other hand, although the SUDs groups in this sample had high rates of cocaine abuse, these participants also used significantly higher rates of alcohol, marijuana, and heroin (Table 2). It is possible that the effects are reflective of polysubstance abuse, rather than a single drug action. Future studies should investigate cocaine addiction without comorbid substances in order to determine the effects of cocaine alone. The value of the current study is that it is likely representative of an inner-city, low-income, and largely ethnic minority population in which high rates of trauma exposure and substance use are frequently observed [48].

## Limitations and Future Directions

Despite the strength of this investigation, results must be considered in light of study limitations. Measurements of all clinical variables were based on client self-report, which can introduce various biases such as social desirability, omission of data, and retrospective bias. Though use of self-report is an accepted means for assessing psychological factors, future studies that employ longitudinal designs are indicated as participant memories could be selective, inconsistent, or otherwise less unreliable [49]. The reading of instruments to subjects by study interviewers instead of receiving written answers from subjects could have also introduced additional response biases [50].

Relying on participant report to diagnose lifetime SUDs poses another study limitation. Although a urine test was administered prior to startle testing and those with positive results were excluded from the study, we do not have reliable information on the duration of abstinence or recent usage by study participants. In addition, research that indicates that acute versus chronic substance use has differential effects on DA levels and other transmitter systems in the brain [51] suggests that startle response could be influenced by whether participants actually have current SUDs.

## Figures and Tables

**Table 1:** Demographic data for the participants in the study across PTSD and SUD groups.

Demographic	CONTROL (N=81)	PTSD ONLY (N=33)	SUD ONLY (N=54)	PTSD+SUD (N=46)
Age, M(SD) years	39.5 (12.8)	34.8 (12.5)	40.0 (11.1)	44.5 (8.3)^b^
Sex, N (%) female	65 (80.2)#	27 (81.8)#	24 (43.6)@	26 (56.5)
Ethnicity, N (%) African American	80 (98.8)#	28 (84.8)	48 (88.9)	43 (93.5)
Employment, N (%) unemployed	55 (67.9)@	29 (87.9)	44 (81.5)	40 (87.0)

Abbreviations: PTSD=Posttraumatic stress disorder; SUDs=Substance Use Disorders

a different from CONTROL group;

b different from PTSD ONLY group;

c different from SUD ONLY group

# greater likelihood;

@ lower likelihood

**Table 2:** Clinical data for PTSD symptoms and lifetime substance abuse across the PTSD and SUD groups.

Measure (M,SD)	Control	PTSD Only	SUD Only	PTSD+SUD
Trauma exposure lifetime (TEI)	3.3 (2.8)	5.7 (3.2)^a^	6.1 (3.1)^a^	8.0 (3.3)^a^,^b^,^c^
PTSD Symptoms (mPSS)	6.9 (7.5)	23.8 (9.6)^a^,^c^	8.5 (6.7)	28.8 (8.2)^a^,^b^,^c^
Re-experiencing Symptoms (mPSS)	1.7 (2.8)	5.9 (3.5)^a^,^c^	1.9 (2.3)	7.0 (3.8)^a^,^c^
Avoidance Symptoms (mPSS)	2.5 (3.4)	9.8 (4.4)^a^,^b^,^c^	3.5 (3.5)	11.9 (3.7)^a^,^b^,^c^
Hyperarousal Symptoms (mPSS)	2.8 (3.1)	8.2 (3.5)^a^,^b^,^c^	3.2 (3.2)	9.8 (3.3)^a^,^b^,^c^
Lifetime Alcohol Use (KMSK)	6.1 (4.4)	6.8 (4.5)	9.9 (2.8)^a^	10.3 (3.1)^a^,^b^
Lifetime Cocaine Use (KMSK)	0.3 (1.4)	1.0 (3.0)	7.1 (6.8)^a^	10.4 (6.1)^a^,^b^,^c^
Lifetime Marijuana Use (KMSK)	2.9 (3.9)	4.8 (4.4)	10.2 (4.3)^a^,^b^	10.0 (4.4)^a^,^b^
Lifetime Heroin Use (KMSK)	0.2 (1.0)	0.1 (0.3)	2.0 (4.1)^a^,^b^,d	0.7 (1.6)

Abbreviations: PTSD=Posttraumatic stress disorder; SUDs=Substance Use Disorders; TEI=Traumatic Experiences Inventory; KMSK=Kreek–McHugh–Schluger–Kellogg scale.

a different from CONTROL group;

b different from PTSD ONLY group;

c different from SUD ONLY group
